# Bacterial Communities and Resistance and Virulence Genes in Hospital and Community Wastewater: Metagenomic Analysis

**DOI:** 10.3390/ijms26052051

**Published:** 2025-02-26

**Authors:** Maria Elena Velazquez-Meza, Miguel Galarde-López, Patricia Cornejo-Juárez, Miriam Bobadilla-del-Valle, Ernestina Godoy-Lozano, Edgar Aguilar-Vera, Berta Alicia Carrillo-Quiroz, Alfredo Ponce de León-Garduño, Consuelo Velazquez Acosta, Celia Mercedes Alpuche-Aranda

**Affiliations:** 1 Centro de Investigación Sobre Enfermedades Infecciosas, Instituto Nacional de Salud Pública, Morelos 62100, Mexico; elizabeth.godoy@insp.mx (E.G.-L.); eavera@insp.mx (E.A.-V.); berta.carrillo@insp.mx (B.A.C.-Q.); 2 Centro Nacional de Investigación Disciplinaria en Salud Animal e Inocuidad, Instituto Nacional de Investigaciones Forestales, Agrícolas y Pecuarias, Cuajimalpa, Mexico City 05110, Mexico; galarde.miguel@inifap.gob.mx; 3 Departamento de Infectología, Instituto Nacional de Cancerología, Tlalpan, Mexico City 14080, Mexico; patcornejo@yahoo.com (P.C.-J.); consueve62@yahoo.com.mx (C.V.A.); 4 Laboratorio Nacional de Máxima Seguridad para el Estudio de Tuberculosis y Enfermedades Emergentes, Instituto Nacional de Ciencias Médicas y Nutrición “Salvador Zubirán”, Mexico City 14080, Mexico; mbv99@hotmail.com (M.B.-d.-V.); alf.poncedeleon@gmail.com (A.P.d.L.-G.)

**Keywords:** metagenomic analysis, resistome, virulome, wastewater, hospital, community

## Abstract

Metagenomic studies have made it possible to deepen the analysis of the abundance of bacterial populations that carry resistance and virulence determinants in the wastewater environment. In this study, a longitudinal collection of samples of community and hospital wastewater from August 2021 to September 2022 was obtained. Shotgun metagenomic sequencing and bioinformatic analysis were performed to characterize the bacterial abundance, antimicrobial resistance genes (ARGs), plasmids, and virulence factor genes (VFGs) contained in the wastewater. The microbial composition of the community and hospital wastewater showed that the most abundant bacterial phyla detected in all samples were: *Proteobacteria*, *Bacteroides*, *Firmicutes*, *Campylobacterota*, and *Actinobacteria*. Seasonal differences in the relative abundances of species, ARGs, plasmids, and VFGs were observed. In this study, a total of 270 ARGs were detected, and it was found that the absolute abundance of ARGs only showed a 39% reduction in the treated wastewater. Furthermore, the ARGs detected in this study were found to encode resistance to antibiotics of the last choice. Our results showed that plasmids carrying resistance genes were more abundant in raw wastewater, and 60% more abundant in hospital wastewater compared to community wastewater. Several of the VFGs detected in this study encode for adhesion, motility, and biofilm formation, which likely allows bacteria to remain and persist in the wastewater environment and survive WWTP treatment systems, thus managing to escape into the environment via treated wastewater.

## 1. Introduction

Antimicrobial resistance (AMR) currently represents one of the most important public health problems, and carries serious health and socioeconomic consequences [1]. Infections caused by resistant bacteria currently cause at least 700,000 deaths per year worldwide. This problem could rise to 10 million per year by 2050 in all income regions. It is estimated that, by 2050, 300 million people will die from AMR if this problem is not overcome [2]. Currently, most of the AMR studies span humans, animals, plants, and the environment, so the detection of bacterial communities, ARGs, plasmids, and GFVs in community and hospital wastewater systems can be an early warning tool for the potential spread of AMR to the environment [3,4].

Wastewater treatment plants (WWTPs) play a crucial role in decreasing the bacterial load of water, and they have been described as hot spots that promote AMR by transferring genetic material [5,6]. Although WWTPs reduce the abundance of bacteria released into the environment, current wastewater treatment processes do not eliminate all antibiotic-resistant bacteria (ARBs), ARGs, plasmids, VFGs, and other determinants such as trace antibiotics, heavy metals, biocides, and detergents. Wastewater monitoring, combined with the use of metagenomics, has been proposed as a tool capable of analyzing the microbiomes of these environments at a deeper level. The analysis of wastewater samples using metagenomics further offers the advantage that it can be used for the relative quantification of a wide variety of bacterial species, ARGs, plasmids, and VFGs present in the sample [7,8]. In this study, wastewater samples were collected from two community and two hospital treatment plants. Three are in Mexico City and the other is in Cuernavaca, Morelos. The community WWTPs have pretreatment and primary and secondary treatment systems; in the hospital WWTPs, one has up to tertiary treatment and the other only pretreatment.

Recent studies have applied metagenomic analysis to identify the resistome in wastewater [9,10]. Pärnänen et al. demonstrated that the ARGs present in wastewater samples collected in several European countries were correlated with resistomes observed in hospitals [9], while Raza et al. found a high abundance of ARGs in effluents from 12 WWTPs in Korea [10]. The viruloma present in wastewater has been addressed in other works, e.g., Mao G et al., who evaluated the role of biofilms as a reservoir and vehicle for virulence factors in wastewater, demonstrating that bacteria in wastewater increased their biofilm production and that the mobility of virulence factors is crucial for biofilm formation [11]. A previous study by our working group evidenced the presence and persistence of bacteria of the *ESKAPEE* group in raw and treated hospital wastewater [12], setting a precedent for metagenomic studies in wastewater in our country and establishing a standard to deepen and broaden the knowledge of AMR in these ecosystems through the longitudinal analysis of community and hospital wastewater samples.

## 2. Results

### 2.1. DNA Extraction and Sequencing

Of the 56 wastewater samples collected during the study period, 31 were sent for metagenomic sequencing; the reason for this selection was that the DNA concentration in several samples was insufficient for metagenomic analysis, in addition to the fact that we were within the budget available for this analysis. The wastewater samples from all the WWTPs studied were considered by their type of water (raw and treated) and seasonality. The average number of readings per sample was 21.4 million readings (range: 12.1 million−31.1 million). The average quality (Q) across all sample bases was 33.6.

### 2.2. Taxonomic Composition of Bacteria in Community and Hospital Wastewater

**Figure 1:**
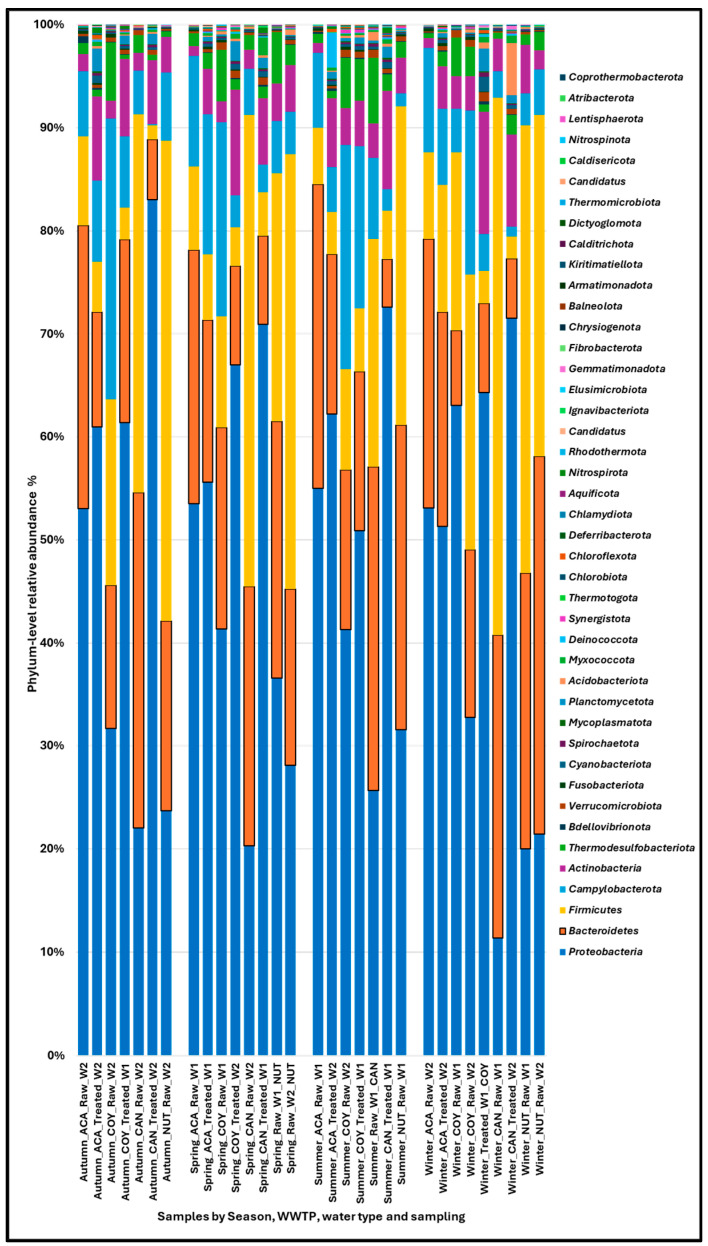
Phylum-level composition of the bacterial population in community and hospital wastewater samples. W1 (sampling 1), W2 (sampling 2).

A total of 43 phyla, 95 classes, 205 orders, 498 families, 1855 genera, and 9276 species were detected in the wastewater samples that were analyzed. The main bacterial phyla detected were: *Protobacteria* (35.03 ± 14.72% raw/64.29 ± 9.40% treated), *Bacteroidetes* (23.78 ± 7.53% raw/11.62 ± 5.28% treated), *Firmicutes* (25.81 ± 15.38% raw/4.69 ± 2.82% treated), *Campylobacterota* (8.74 ± 7.16% raw/5.70 ± 4.87% treated), and *Actinobacteria* (2.63 ± 1.17% raw/7.36 ± 2.48% treated). The relative abundance per phylum in the community WWTPs was as follows: *Protobacteria* (47.20 ± 20.82% raw/59.19 ± 5.96% treated), *Bacteroidetes* (20.01 ± 7.42% raw/14.32 ± 4.2% treated), *Firmicutes* (12.58 ± 6.76% raw/5.49 ± 3.04% treated), *Campylobacterota* (13.61 ± 7.80% raw/7.82 ± 4.63% treated), and *Actinobacteria* (2.03 ± 1.06% raw/7.17 ± 2.88% treated). The values for the hospital WWTP were as follows: *Protobacteria* (24.08 ± 6.94% raw/74.50 ± 5.73% treated), *Bacteroidetes* (27.18 ± 6.10% raw/6.22 ± 1.66% treated), *Firmicutes* (37.72 ± 10.01% raw/3.11 ± 1.61% treated), *Campylobacterota* (4.36 ± 1.88% raw/1.47 ± 1.13% treated), and *Actinobacteria* (3.16 ± 1.04% raw/7.75 ± 1.71% treated) (Figure 1).

The seasonal distribution of the relative abundance of the phylum *Protobacteria* was very similar in all seasons and ranged from 48.45 ± 16.71% in summer to 43.20 ± 22.18% in winter; the distribution of the relative abundance by WWTP showed that the community WWTPs ACA and COY had the highest abundance of the phylum *Protobacteria*. The results by type of wastewater (raw and treated) showed that the relative abundance of the phylum *Protobacteria* was higher in treated wastewater: 61.89 ± 10.86% in summer; 62.36 ± 10.24% in winter; 64.47 ± 7.96% in spring and 68.45 ± 12.62% in autumn.

For the phylum *Bacteroidetes*, the relative abundance by season was very similar, fluctuating between 20.22 ± 10.06% in summer and 18.13 ± 9.25% in autumn; the analysis by WWTP showed that the relative abundance was homogeneous within each WWTP, but different between WWTPs, being higher in the hospital WWTPs (CAN and NUT). For this phylum, the relative abundance by wastewater type was higher in raw wastewater: 22.27 ± 3.69% in spring; 23.06 ± 8.48% in autumn; 23.73 ± 10.42% in winter; and 26.48 ± 7.40% in summer.

For the phylum *Firmicutes* it was observed that the relative abundance by season was higher in winter, 22.09 ± 17.88%, lower in summer, 11.88 ± 10.48%, and very similar in spring and autumn. The relative abundance within each WWTP was very similar except in the ACA WWTP, where, in winter, this abundance increased (52.19 ± 0.29%) compared to the values observed in spring, summer, and autumn. The results of the relative abundance by wastewater type showed that, for this phylum, it was higher in raw wastewater: 17.08 ± 1.62% in summer, 26.19 ± 17.39% in spring, 27.51 ± 17.29% in autumn, and 30.18 ± 16.22% in winter.

For the phylum *Actinobacteria*, the seasonal relative abundance was homogeneous, fluctuating between 4.24 ± 2.99% in spring and 4.67 ± 3.50% in winter. This phylum presented a higher relative abundance in treated wastewater: 6.87 ± 2.57% in summer, 7.01 ± 3.00% in spring, 7.28 ± 0.02% in autumn, and 8.30 ± 3.91% in winter; in general, for this phylum, the relative abundance per WWTP was lower compared to the results observed for *Protobacteria*, *Bacteroidetes*, and *Firmicutes*. All results of relative abundance by phylum are shown in Supplementary Materials Table S1.

Of the 1855 bacterial genera detected in the wastewater samples that were analyzed, it was observed that 114 genera presented the highest relative abundances in almost all the stations and WWTPs studied, including *Acidovorax*, *Acinetobacter*, *Aeromonas*, *Aliarcobacter*, *Arcobacter*, *Bacteroides*, *Blautia*, *Burkholderia*, *Chryseobacterium*, *Cloacibacterium*, *Clostridiodes*, *Comamonas*, *Coprococcus*, *Enterobacter*, *Escherichia*, *Faecalibacterium*, *Flavobacterium*, *Hydrogenophaga*, *Klebsiella*, *Parabacteroides*, *Phocaeicola*, *Prevotella*, *Pseudomonas*, *Streptococcus*, *Streptomyces*, *Sulfurospirillum*, *Thauera*, *Tolumonas*, and *Vibrio*. (Supplementary Materials Table S2). In this study, 9276 species were detected, of which 110 showed the highest relative abundances in almost all the stations and WWTPs studied, including *Acidovorax temperans*, *Acinetobacter johnsonii*, *Acinetobacter baumannii*, *Aeromonas caviae*, *Aeromonas hydrophila*, *Bacteroides fragilis*, *Acinetobacter johnsonii*, and *Pseudomonas aeruginosa*, among others (Supplementary Materials Table S3).

The relative abundance of eight bacterial species included in the *ESCKAPEE* group (*Enterococcus faecium*, *Staphylococcus aureus*, *Clostridioides difficile*, *Klebsiella pneumoniae*, *Acinetobacter baumannii*, *Pseudomonas aeruginosa*, *Enterobacter cloacae*, and *Escherichia coli*) was analyzed, considering that these species are clinically relevant.

The results of the relative abundance of the ESCKAPEE-group bacteria detected in the community and hospital wastewater showed that the highest abundances were found in *E. coli* (0.51 ± 0.45%), *A. baumannii* (0.25 ± 0.26%), *P. aeruginosa* (0.22 ± 0.10%), and *K. pneumoniae* (0.10 ± 0.07%), while, for *E. cloacae* and the Gram-positive species, the relative abundance was lower.

**Figure 2:**
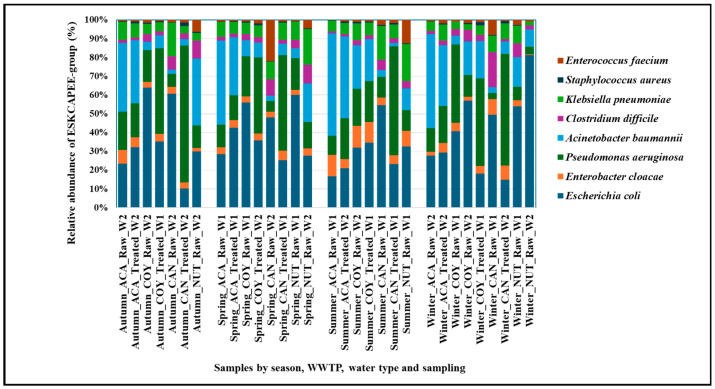
Relative abundance of *ESCKAPEE*-group bacteria at the species level from both community and hospitals wastewater.

The seasonal distribution of the relative abundance by species showed that, for *E. faecium*, it was higher in spring (0.06 ± 0.12%) and summer (0.04 ± 0.08%); while, for *C. difficile*, no marked seasonal differences were observed. *S. aureus* remained the same in all seasons (0.01 ± 0.01%). The seasonal distribution of the relative abundance of the Gram-negative bacteria was higher in summer, except for that of *E. coli*, which presented high values in all seasons, fluctuating between 0.39 ± 0.17% in summer and 0.63 ± 0.74% in winter (Figure 2).

The relative abundance by species between the WWTPs showed that the wastewater from the hospital WWTPs (CAN and NUT) had a higher abundance of *E. faecium* (CAN 0.06 ± 0.12%/NUT 0.07 ± 0.08%) and *C. difficile* (CAN 0.06 ± 0.06%/NUT 0.09 ± 0.02%) compared to the wastewater from the community WWTPs (ACA and COY), and that the relative abundance of *S. aureus* was similar in all WWTPs (0.01 ± 0.01%). In wastewater from the community WWTPs (ACA and COY), *A. baumannii*, *P. aeruginosa*, and *E. coli* were more abundant, while *K. pneumoniae* presented similar abundances, except in the samples from the COY WWTP, which had a lower abundance (0.05 ± 0.02%).

The relative abundance of these species according to the type of wastewater (raw/treated) showed that, in raw wastewater, this abundance was higher, except for *S. aureus*, for which it was similar (0.01 ± 0.01%), and for *P. aeruginosa*, where the relative abundance was higher in the treated wastewater (0.26 ± 0.06%) (Figure 2).

**Figure 3:**
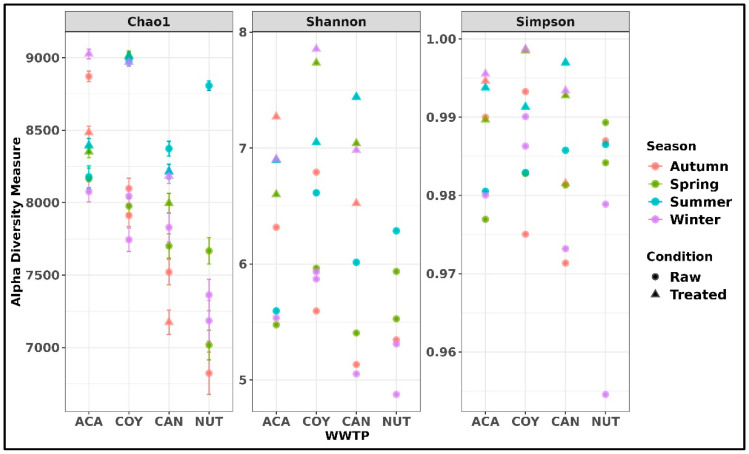
Alpha diversity indexes of each treatment at the species level, Chao1 diversity index, Shannon diversity index, and Simpson diversity index.

From the taxonomic annotations at the species level, the alpha diversity was determined with a value of *p* < 0.05 using three indices. The mean richness observed using the chao1 index in the analyzed wastewater samples was 8102 ± 619.77 unique species, showing a different richness between treatments and between each WWTP (8000–8500 ACA, 7750–8250 COY, 7400–8400 CAN, and 6250–7750 NUT), with an inter quartile interval (IIQ) of 7723–8440; in relation to seasonal abundance, a higher richness was observed in spring and summer. The Shannon index showed a higher richness of bacterial species in the treated wastewater samples (6.5–7.8), indicating that the bacterial diversity of the raw and treated wastewater samples was in the range of 4.87–7.85, with an IIQ 5.530–6.905. Simpson’s alpha diversity index showed that the most diverse bacterial communities were those of the raw wastewater samples (0.95–0.99), compared to the treated wastewater samples (0.99–1.00) (Figure 3).

**Figure 4:**
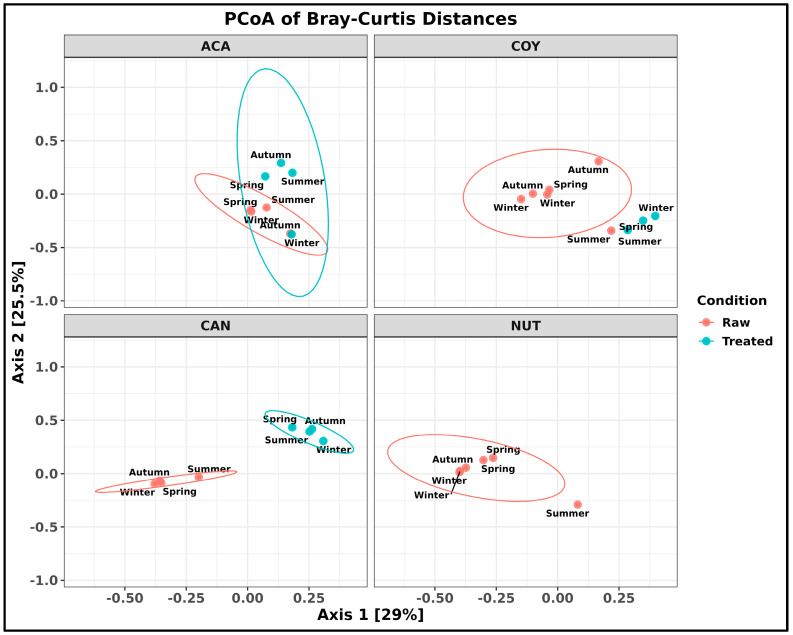
Analysis of similarity (ANOSIM). Principal coordinate analysis between wastewater samples using the Bray–Curtis distance matrix at the species level.

A principal coordinate analysis (PCoA) was performed at the species level to determine the type of wastewater condition (raw and treated), the relationship between samples at the species level, and the sampling time for each sample. In this analysis, it was observed that the wastewater samples from the CAN WWTP were perfectly grouped according to wastewater type (raw and treated), but this was not the case for the samples from the ACA and COY WWTPs, where a treated wastewater sample overlapped within the universe of raw wastewater samples. Despite this occurrence, the results suggest differences in species composition between the two types of wastewaters that were analyzed. Between sampling time intervals in the treated wastewater, stational differences in the bacterial species composition were observed (*p*-value < 0.05). In the comparison of the wastewater samples, principal components 1 and 2 explained 54.5% of the variation after the samples underwent the processes in the WWTPs (Figure 4).

### 2.3. Analysis of Antimicrobial Resistance Genes

The CARD database was used to determine the presence, identity, relative abundance, absolute abundance, and persistence of ARGs in each sample and at each station. A total of 270 ARGs were detected in the wastewater samples that were analyzed, showing an absolute abundance of 95.1 ± 20.9 in the raw wastewater and 56.5 ± 25.8 in the treated wastewater. The absolute abundance of ARGs by WWTP and wastewater type was as follows: communities: ACA 98 ± 11.2 raw/70.3 ± 28.7 treated; COY 99.3 ± 18.6 raw/55.5 ± 33.7 treated; hospitals: CAN 99.5 ± 19.9 raw/43.7 ± 4.4 treated; NUT 83.7 ± 33.1 raw. The seasons where the highest abundances of ARGs were detected were spring and summer, coinciding with the seasons with the highest abundances of bacterial species.

In the wastewater samples analyzed in this study, the *qacH*, *aadS*, *InuD*, *APH(3′)-IIIa*, *catB3*, *msrE*, and *OXA-2* genes were the most frequent. The number of ARGs detected by WWTP varied: ACA n = 162, COY n = 182, CAN n = 185, and NUT n = 163. The number of ARGs present at all stations was different at each WWTP: **ACA/n** = **23** (*qacH*, *catB3*, *msrE*, *OXA-2*, *mphA*, *tet(C)*, *mphE*, *QnrVC1*, *aadA2*, *APH(3″)-Ib*, *dfrA1*, *dfrA15*, *ANT(3″)-IIa*, *OXA-10*, *OXA-427*, *tet(A)*, *tet(39)*, *aadA11*, *ErmG*, *QnrB5*, *QnrS2*, *QnrVC4*, and *tet(E)*; **COY/n** = **10** (*qacH*, *catB3*, *msrE*, *OXA-2*, *mphA*, *QnrVC1*, *APH(3″)-Ib*, *dfrA15*, *tet(X)*, and *ANT(3″)-IIa*); **CAN/n** = **12** (*qacH*, *catB3*, *msrE*, *OXA-2*, *cmlA5*, *tet(C)*, *dfrA1*, *sul1*, *TLA-1*, *AAC(6′)-Il*, *sul2*, and *floR*), and **NUT/n** = **28** (*qacH*, *aadS*, *APH(3′)-IIIa*, *catB3*, *msrE*, *OXA-2*, *cmlA5*, *mphA*, *tet(C)*, *APH(6)-Id*, *mphE*, *QnrVC1*, *TLA-2*, *aadA2*, *APH(3″)-Ib*, *dfrA15*, *OXA-427*, *tet(A)*, *APH(3′)-Ia*, *AAC(6′)-33*, *AAC(6′)-Il*, *dfrA12*, *ErmG*, *Mef(En2)*, *aadA10*, *OXA-58*, *tet(40)*, and *OXA-372*). The seasonal distribution, persistence, and frequency of the ARGs detected in the community and hospital wastewater samples that were studied are shown in Supplementary Materials Figures S1 and S2.

**Figure 5:**
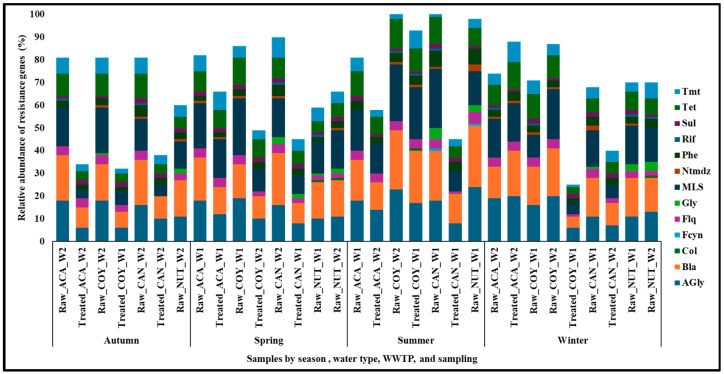
Relative abundance of resistance genes encoding antibiotic classes in wastewater samples. Drug classes: Agly (aminoglycoside), Bla (betalactam), Col (colistina), Fcyn (fosfomycin), Flq (fluoroquinolana), Gly (glycopeptides), MLS (macrolides–lincosamides–streptogramines), Ntmdz (nitroimidazole), Phe (phenicol), Rif (rifampicin), Sul (sulfonamides), Tet (tetracycline), and Tmt (trimethoprim).

The most abundant and persistent ARGs detected in the wastewater samples analyzed were those that confer resistance to aminoglycosides (*APH(3′)-IIIa*, *APH(3″)-Ib*, *APH(6)-Id*, *APH(3′)-Ia*, *aadA2*, *aadA11*, *aadA10*, *AAC(6′)-Il*, *AAC(6′)-33*, and *ANT(3″)-IIa*), beta-lactams (*OXA-2*, *OXA-10*, *OXA-427*, *TLA-1*, *TLA-2*, *OXA-58*, and *OXA-372*), macrolides lincosamines and streptogramins type B (MLS) (*mphA-E*, *msrE*, *Mef(En2*), *ErmG,* and *InuC-D*), and tetracyclines (*tet(X)*, *tet(C)*, *tet(A)*, *tet(39)*, *tet(E)*, and *tet(40)*). Genes conferring resistance to other groups of antimicrobials: quinolones (*QnrVC*, *QnrVC1*, *QnrB5*, *QnrS2*, and *QnrVC4*), polymyxins (*mcr*-3.3, and *mcr*-5), glycopeptides (*vanUG*, *vanXYG*, *vanTG*, *vanSB*, *vanB*, *vanHB*, *vanRB*, *vanWB*, *vanWG*, *vanXB*,and *vanG*), and others, were found in lower abundance (Figure 5 and Supplementary Materials Figures S1 and S2).

The PlasmidFinder database was used to determine the presence, identity, absolute abundance, and persistence of plasmids carrying ARGs. A total of 60 plasmids were detected in the studied samples of raw (39.1 ± 23.4) and treated (12.8 ± 8.4) wastewater collected from community and hospital WWTPs. The absolute abundances of plasmids per WWTP and wastewater type were as follows: community: ACA 38.2 ± 19.1 raw/20.5 ± 7.7 treated and COY 22.2 ± 4.8 raw/11.0 ± 7.6 treated; hospital: CAN 59.2 ± 14.8 raw/7.0 ± 4.1 treated and NUT 37.0 ± 34.7 raw. The seasons with the highest detected plasmid abundances were spring and summer.

The most abundant plasmids detected in the wastewater samples analyzed were: *Col440I_1*, *ColRNAI_1*, *Col440II_1*, *ColKP3_1*, *IncQ2_1*, *ColE10_1*, *IncQ1_1*, *repUS2_1_repA(pBI143)*, and *Col156_1*. These plasmids carry different ARGs: ***Col440I_1*** carries *NDM-1*, *bla*ampH, *bla*CTX-M-15, and *bla*TEM-1; ***ColRNAI_1*** carries *fosA*; ***Col440II_1*** carries *qnrB*, *cmlA1*, and *fosA7*; ***ColKP3_1*** carries *bla*OXA-131 and *bla*OXA-232; ***IncQ2_1*** and ***IncQ1_1-2*** carry *qnrB77*, *qnrB2*, *qnrS1*, *qnrS2*, and *aac-(6)-lb′-cr*; and ***repUS2_1_repA(pBI143)*** carryies *optrA*. The number of plasmids detected per WWTP varied: ACA n = 34, COY n = 32, CAN n = 45, and NUT n = 45. The number of plasmids present at all stations was different in each WWTP: **ACA/n** = **13** (*Col440I_1*, *ColRNAI_1*, *Col440II_1*, *ColKP3_1*, *IncQ2_1*, *IncQ1_1*, *ColE10_1*, *IncA/C2_1*, *Col156_1*, *IncP6_1*, *IncP6_1*, *repUS2_1_repA(pBI143)*, *IncFIB(K)_1_Kpn3*, and *IncU_1*); **COY/n** = **11** (*IncQ2_1*, *ColRNAI_1*, *Col440I_1*, *ColKP3_1*, *repUS2_1_repA(pBI143)*, *Col156_1*, *IncP6_1*, *IncQ1_1*, *Col(MG828)_1*, *Col440II_1*, and *IncA/C2_1*); **CAN/n** = **15** (*ColRNAI_1*, *Col440I_1*, *Col440II_1*, *ColKP3_1*, *ColE10_1*, *IncQ2_1*, *Col(MG828)_1*, *Col8282_1*, *IncA/C2_1*, *IncFIA(HI1)_1_HI1*, *repUS2_1_repA(pBI143)*, *ColpVC_1*, *IncFIB(K)_1_Kpn3*, and *IncFIB(pB171)_1_pB171*); and **NUT/n** = **8** (*Col440I_1*, *ColRNAI_1*, *IncQ2_1*, *Col440II_1*, *Col156_1*, *ColKP3_1*, *repUS2_1_repA(pBI143)*, and *IncU_1*) (Supplementary Materials Figures S3 and S4).

The plasmids that persisted in the raw and treated wastewater, compared between each sampling (W1 and W2) and each station, varied by WWTP: **ACA/n** = **17** (*Col440I_1*, *ColRNAI_1*, *Col440II_1*, *ColKP3_1*, *IncQ2_1*, *IncQ1_1*, *ColE10_1*, *IncA/C2_1*, *Col156_1*, *IncP6_1*, *IncP6_1*, *repUS2_1_repA(pBI143)*, *IncFIB(K)_1_Kpn3*, *IncU_1*, *Col(MG828)_1*, *Col(MGD2)_1*, *Col8282_1*, and *ColpVC_1*); **COY/n** = **3** (*IncQ2_1*, *Col440I_1*, and *repUS2_1_repA(pBI143*); **CAN/n** = **9** (*Col440I_1*, *Col440II_1*, *ColKP3_1*, *ColE10_1*, *IncQ2_1*, *IncA/C2_1*, *IncFIA(HI1)_1_HI1*, *IncP6_1*, and *repA_2_pKPC-2*). In NUT this persistence was not estimated because only raw water samples were available (Supplementary Materials Figures S3 and S4).

### 2.4. Analysis of Virulence Factor Genes

The virulence factor database (VFDB) was used to determine the presence, identity, absolute abundance, and persistence of genes that encode VFGs. A total of 149 virulence factor genes were detected in the wastewater samples collected from community and hospital WWTPs: raw: 36.1 ± 32.8; and treated: 17.4 ± 15.7. The absolute frequency of plasmids per WWTP and wastewater type was as follows: community: ACA 43.7 ± 20.9 raw/24.5 ± 19.1 treated and COY 50.2 ± 45.1 raw/24.0 ± 12.8 treated; hospital: CAN 16.0 ± 10.1 raw/3.7 ± 2.5 treated and NUT 34.2 ± 44.1 raw (Supplementary Materials Figures S5 and S6).

The virulence genes *pilG*, *pilH*, *pilT*, *xcpT*, *flG*, *algU*, *csgB*, and *csgF* were the most abundant in the wastewater samples analyzed; the *pilG*, *pilH*, *pilT*, and *xcpT* genes are associated with adhesion and motility in several enterobacteria; *flG* is present in *S. aureus* and is associated with alteration of the cutaneous microbiome and cutaneous immune response; *algU* is involved in alginate synthesis, producing an alginate capsule in *P. auruginosa*, which favors its pathogenicity; and *csgB* and *csgF* are associated with biofilm formation in enterobacteria. The number of VFGs varied in each WWTP: ACA n = 89, COY n = 83, CAN n = 39, and NUT n = 103. The number of VFGs that persisted across seasons was different in each WWTP: **ACA/n** = **10** (*pilG*, *pilT*, *pilH*, *algU*, *hsiB1/vipA*, *algR*, *hsiC1/vipB*, *algW*, *fiml*, and *pilU*); **COY/n** = **4** (*pilG*, *pilT*, *xcpT*, and *csgB*); **CAN/n** = **1** (*csgB*); in NUT no seasonal persistence was observed (Supplementary Materials Figures S5 and S6).

The VFGs that persisted in the raw and treated wastewater, compared between each sampling (W1 and W2) and each station, varied by WWTP: **ACA/n** = **38** (*pilG*, *pilT*, *pilH*, *algU*, *hsiB1/vipA*, *algR*, *flgC*, *fliP*, *hsiC1/vipB*, *algW*, *fimI*, *fleN*, *fliN*, *waaF*, *yagZ/ecpA*, *algC*, *csgF*, *fepC*, *fimC*, *fimH*, *flgG*, *fliR*, *pilI*, *xcpT*, *csgB*, *fleQ*, *gspI*, *xcpS*, *yagV/ecpE*, *ykgK/ecpR*, *algA*, *espR1*, *flgI*, *fliE*, *fliG*, *gspJ*, *gspL*, *motA*, and *motC*); **COY/n** = **3** (*pilG, pilT*, and *xcpT*); **CAN/n** = **1** (*pilT*); in NUT this persistence was not estimated because only raw water samples were available (Supplementary Materials Figures S5 and S6).

## 3. Discussion

Currently, the study of wastewater has attracted considerable interest, especially as a model for AMR surveillance, since water is a medium through which ARBs, ARGs, plasmids, and VFGs are disseminated from community and hospital settings to the environment. To this end, metagenomic studies have been crucial in detecting the presence, identity, abundance, and persistence of ARGs, plasmids, and VFGs in bacterial populations present in the wastewater environment. In this study, the most abundant phyla were *Protobacteria*, *Bacteroidetes*, *Firmicutes*, and *Actinobacteria* (Figure 1); the predominance of these phyla in community and hospital wastewater are the same as those described before [12,13].

### 3.1. Taxonomic Composition of Bacteria in Community and Hospital Wastewater

The microbial composition of the hospital and community wastewater at the genus level showed that, within the genera presenting the highest relative abundances, several genera associated with human and animal infections were found: *Acinetobacter*, *Aeromonas*, *Burkholderia*, *Clostridiodes*, *Enterobacter*, *Escherichia*, *Flavobacterium*, *Klebsiella*, *Pseudomonas*, *Streptococcus*, *Streptomyces*, and *Vibrio*, as well as those related to intestinal microbiota: *Bacteroides*, *Blautia*, *Cloacibacterium*, *Comamonas*, *Coprococcus*, *Escherichia*, *Faecalibacterium*, *Parabacteroides*, *Phocaeicola*, and *Prevotella,* and those found in soil and water: *Chryseobacterium*, *Flavobacterium*, *Hydrogenophaga*, *Sulfurospirillum*, *Thauera*, and *Tolumonas* (Supplementary Materials Table S2). The presence of these genera has been reported in other studies, i.e., Lu X et al., who reported high abundances of *Aeromonas*, *Arcobacter*, *Clostridiodes*, and *Pseudomonas* in wastewater samples [14]; Poopedi E et al. detected high abundances of the genera *Escherichia*, *Shigella*, *Arcobacter*, *Acinetobacter*, *Streptococcus*, and *Aeromonas* [15]. The abundant presence of bacterial genera associated with the intestinal microbiota in wastewater samples was reported by Zhang D et al., who detected, in their samples, *Bacteroides*, *Faecalibacterium*, *Parabacteroides*, and *Prevotella* [16].

The microbial compositions of the hospital and community wastewater at the species level showed that, within the species that had the highest relative abundances, several medically important species were found *Escherichia coli*, *Enterobacter cloacae*, *Klebsiella pneumoniae*, *Pseudomonas aeruginosa*, *Acinetobacter baumannii*, *Clostridioides difficile*, and *Acidovorax temperans*, among others (Supplementary Materials Table S3). Some of these species were also reported by Lepper H et al., who detected *Flavobacterium* spp., *Acinetobacter, Wuhouensis*, *Kinneretia*, *Asaccharophila*, *Acidovorax*, *Konjaci*, and *Pseudomonas* spp. as the most abundant species in the wastewater samples analyzed in their study [17]. Furthermore, Fresia P et al. detected significant abundances of *Klebsiella*, *Pseudomonas*, *Acinetobacter*, *Enterobacter*, and *Streptococcus* in wastewater samples [18].

Bacteria of the *ESCKAPEE* group are included within the phyla *Proteobacteria* and *Firmicutes*; these phyla have been reported in other studies as the most abundant [12,19,20]. Our results showed that *E. coli*, *A. baumannii*, *P. aeruginosa*, and *K. pneumoniae* were the most abundant species, with all having a marked seasonality in summer except for *E. coli*, which presented high abundances in all seasons. For *E. faecium*, the relative abundance was higher in spring and summer, while, for *C. difficile*, no marked seasonal changes were observed, and, for *S. aureus*, the abundance remained the same in all seasons (Figure 2). The seasonal distribution of some microorganisms has been pointed out in other works, in which several contributing factors are highlighted. He Y et al. found that the temperature and chemical oxygen demand were the main environmental factors affecting the bacterial community structure of the wastewater that they tested, with temperature having the most significant effect on the species composition [21]. In another work, Kang et al. reported that the diversity and relative abundance of bacterial communities in wastewater collected in winter were significantly lower compared to summer samples, and that the temperature and dissolved oxygen were the main factors driving seasonal changes in the bacterial diversity, richness, and community structure in the WWTPs that they studied [22].

The relative abundance by species between WWTPs showed that the wastewater of CAN and NUT had a higher abundance of *E. faecium* and *C. difficile* than the wastewater from ACA and COY. The abundance of *E. faecium* and *C. difficile* in the hospital wastewater could be related to the hospital origin of these species. Sanderson H et al. mentioned that *E. faecium* may be more adapted to the clinical environment than to the wastewater environment [23], while Moradigaravand D et al. found that clinical strains of *C. difficile* were closely related to isolates present in hospital wastewater [24]. The relative abundance of *S. aureus* remained the same in the wastewater samples collected from all WWTPs, similar to what we observed before, with no differences between the frequency of staphylococcal isolates in community wastewater compared to hospital wastewater [25]; this may demonstrate the ubiquitous capacity of this species to remain in both environments. The same behavior was observed for *K. pneumoniae*, demonstrating the ability of this species to stay in both community and hospital wastewater. *A. baumannii*, *P. aeruginosa*, and *E. coli* were more abundant in the wastewater from the community WWTPs (ACA and COY) (Figure 2). Two of these species (*E. coli* and *P. aeruginosa*) were reported by Galarde L et al. as the most abundant in hospital wastewater [12], while Hubeny J et al. reported that *A. baumannii* and *E. coli* were the dominant species in community wastewater [26], species that we also detected in the community wastewater of this study.

When the relative abundances of the *ESCKAPEE*-group bacteria were compared by type of wastewater (raw/treated), it was observed that *E. faecium*, *C. difficile*, *E. coli*, *A. baumannii*, and *K. pneumoniae* were more abundant in the raw wastewater, and the high abundance of these species in raw wastewater samples has been associated with human feces [27,28]. While *S. aureus* remained constant, for *P. aeruginosa*, the relative abundance was higher in treated wastewater (Figure 2). The ability of this species to increase in abundance in treated wastewater is probably related to its ability to form biofilms [29]. The abundance of species of this group in wastewater was also described by our work group, with *E. coli*, *Klebsiella* spp., and *Enterobacter* spp. being the predominant genera in the raw wastewater, while *Enterococcus* spp., *Staphylococcus* spp., *Acinetobacter* spp., and *Pseudomonas* spp. being more abundant in the treated wastewater [12].

Principal coordinate analysis (PCoA) showed that the wastewater samples from the CAN WWTP were perfectly grouped according to wastewater type (raw and treated), but this was not the case for the samples from the ACA and COY WWTPs, where a treated wastewater sample overlapped with raw wastewater samples (Figure 4). According to the records of each sampling, it was documented that, in these collections, the ACA and COY WWTPs had mechanical failures in pumping, which probably impacted the correct treatment of raw wastewater, resulting in an output of untreated wastewater, which would explain this result; the discharge of untreated wastewater can have an ecological impact when it is reused due to its potential for the dissemination of ARBs and ARGs in the environment.

### 3.2. Analysis of Antimicrobial Resistance Genes

Wastewater is increasingly recognized as an important reservoir of ARGs, allowing conditions that are conducive to selection pressure and the horizontal transfer of resistance genes (HTGs). Wastewater is rich in partially metabolized antibiotics, heavy metals, detergents, and biocides. Together, these compounds can exert selective pressure on the proliferation of ARBs. The high microbial load of these environments may be an ideal component for the proliferation of ARBs through the HTG process [30]. Furthermore, Karkman A et al. and Pilmis B et al. report that the human and animal gut microbiomes contain a wide variety of ARBs; thus, wastewater discharge and fecal contamination have been associated with increased abundances of ARBs in the aquatic environment [31,32]. In this study, a total of 270 ARGs were detected, and it was observed that the absolute abundance of ARGs showed only a 39% reduction in treated wastewater; but this reduction seems to vary in different studies, as some have reported a 70% reduction of ARGs in treated hospital wastewater [12], while Yang Y. et al. and Gupta S. et al., reported that wastewater treatment removed more than 99% of ARGs in community WWTPs in Hong Kong and South Korea, respectively [33,34], and Szczepanowski R et al. reported a reduction of 13% in treated wastewater from a WWTP in Germany [35]. The differences in the reduction percentages of each WWTP could probably be related to the types of treatment within each plant.

When comparing the absolute abundances of ARGs from community WWTPs (ACA/COY) versus hospital WWTPs (CAN/NUT), it was observed that, regardless of the WWTP, the abundances of ARGs were very similar in the raw wastewater samples, (ACA 98 ± 11.2, COY 99.3 ± 18.6, CAN 99.5 ± 19.9, and NUT 83.7 ± 33.1. These results are interesting because hospital WWTPs have been documented to contribute a higher load of ARGs to wastewater compared to community WWTPs due to antibiotic use within hospitals, the discharge of antibiotic residues to hospital effluent, and trace antibiotics in patient excreta [36,37]. However, our results show that, in community wastewater, ARGs are also abundant, which could possibly reflect the consumption of antibiotics in the community or the arrival of ARGs to community WWTPs through wastewater discharges from private health clinics.

The reduction in ARGs abundance by WWTPs was 28% in ACA, 43.8% in COY, and 55.8% in CAN, and it was observed that the hospital WWTP (CAN) had a higher reduction of ARGs, i.e., the abundance of ARGs in the treated wastewater from this WWTP was lower (43.7 ± 4.4) compared to that detected in ACA (70.3 ± 28. 7) and COY (55.5 ± 33.7). One explanation for this result may be the fact that the CAN WWTP has a tertiary treatment system, which would have favored the reduction of ARGs, while the community WWTPs (ACA and COY) both only have a secondary treatment system.

Our results showed that the *qacH*, *aadS*, *InuD*, *aph(3′)-IIIa*, *catB3*, *msrE*, and *bla*OXA genes were the most abundant in the wastewater samples analyzed (Supplementary Materials Figures S1 and S2). Some of these genes were previously reported with high abundance and persistence in wastewater samples from two hospital WWTPs in Mexico [12]. The *qacH* gene has been documented to encode for resistance to quaternary ammonium salts, heavy metal, and disinfectants and has been detected in several bacterial species (*Staphylococcus* spp. *E. faecalis*, *E. coli*, *Listeria monocytogenes*, *Klebsiella*, *Enterobacter*, *Citrobacter*, and *Pseudomonas*) [38,39,40,41,42,43].

The *aadS* gene confers resistance to aminoglycosides and has been described in *Elizabethkingia meningoseptica* [44] and in *Chryseobacteria.* Pham D. et al. found that *aadS* is found in mobile genetic elements, suggesting its high transmissibility across the phylum *Bacteroidetes*, including its transfer between different species [45]. The *aph(3′)-IIIa* gene encodes aminoglycoside resistance and has been described in methicillin-resistant *S. aureus* strains isolated from hospital wastewater in Portugal [46], as well as in *Enterococcus* spp. isolated from various sources, including wastewater [47,48]. Other ARGs were detected that also encode aminoglycoside resistance were detected in this study, including *aads*, *aadA2*, and *aac3*, which have been reported in other work [9,10,12].

The *catB3* gene, which confers resistance to chloramphenicol, has been detected in *Aeromonas*, *Bordetella*, *Pseudomonas*, and *Enterorobacteriaceae* and is considered an abundant gene in wastewater [49,50]. Other genes abundantly detected in our study were *msrE*, *mph*(A, E, G), *mef*(B,C), *Inu*(C,D), and *erm*(B,F,G), which encode resistance to MLS. Pallares-Vega R et al. detected these same genes in wastewater from 62 WWTPs in the Netherlands [51]. The *erm*(B) gene was originally detected in Gram-positive bacteria (*Enterococcus* spp., *Staphylococcus* spp., and *Streptococcus* spp.), and this gene can be transferred to Gram-negative bacteria through a conjugative transposon [9,52]. In our study, the *bla*OXA genes were widely represented. All these genes have been detected in hospital and community wastewater from several countries [12,49,53,54,55,56,57]. Ramos et al. observed that the diversity and abundance of ARGs were not limited to the *ESKAPE* group, but that these genes predominated in bacteria such as *Aeromonas*, *Aliarcobacter*, and *Acidovorax*, finding that *Aliarcobacter* accumulated a high abundance of genes for resistance to sulfonamides and polymyxins, while *Acinetobacter* and *Aeromonas* harbored the highest abundances of ARGs against beta-lactams [58]. These four species were also abundant in our study, which may imply important reservoirs of ARGs in the analyzed wastewater (Supplementary Materials Tables S1 and S2).

It was observed that the ARGs detected in this study in hospital and community wastewater encode resistance to surveillance and reserve antibiotics, according to the WHO AWaRe classification [59]. These included carbapenems (Imipenem) (*bla*OXA-58, *bla*OXA-372, and *bla*-OXA-427); aminoglycosides (kanamycin, neomycin, streptomycin, ribostamycin), (*aph(3″-IIIa, aph(3″)-Ib, aph(3″)-Ia, ant(3″)-IIa, aac(6″)-Il*, *aac(6″)-33, aadA2*, *aadA11*, and *aadA10*); and the MLS group (erythromycin, ciprofloxacin, clindamycin), (*mphA*, *mphE msrE*, *Mef(En2*), and *ErmG*). All these genes have been detected in wastewater in other studies [60,61,62,63,64,65,66,67,68,69,70]. Also, genes encoding resistance to quinolones (ciprofloxacin), polymyxins (colistin), and glycopeptides (vancomycin) were also detected (Figure 5 and Supplementary Materials Figures S1 and S2). In this study, the *mcr*-5 and *mcr*-3.3 genes were detected in raw wastewater from NUT and treated wastewater from COY, respectively (Supplementary Materials Figures S1 and S2). The *mcr* gene was not previously detected in hospital wastewater in Mexico [12]; however, it has been detected in community wastewater in other countries, including Spain, France, Germany, and Tunisia [55,71,72,73]. The presence of genes encoding resistance to antibiotics of the last choice in hospital and community wastewater in our country represents a problem that requires surveillance.

Our results showed that plasmids carrying resistance genes were more abundant in raw wastewater, and 60% more abundant in hospital wastewater compared to community wastewater. *Col440I_1*, *ColRNAI_1*, *Col440II_1*, *ColKP3_1*, *IncQ2_1*, *ColE10_1*, *IncQ1_1*, *repUS2_1_repA(pBI143)*, and *Col156_1* were the most abundant plasmids (Supplementary Materials Figures S3 and S4). These plasmids carry genes encoding resistance to beta-lactams, quinolones, fosfomycin, phenicols, oxazilidones, lincosamides, aminoglycosides, sulfonamides, and tetracyclines. The study by Chukamnerd A et al. identified a high frequency of *Col440I* and *ColKP3* plasmids in *K. pneumoniae* [74], while the works by Bönemann, G et al., Loftie-Eaton W et al., and Piotrowska M et al. demonstrated that IncQ-group plasmids carry several ARGs, which confer resistance to beta-lactams, aminoglycosides, carbapenems, phenicols, lincosamides, quinolones, tetracyclines, and sulfonamides [75,76,77]. Importantly, the presence of plasmids carrying ARGs in community and hospital wastewater favors the spread of antimicrobial resistance and enhances the transfer of resistance between bacteria of the same or different genera, representing an important public health problem.

### 3.3. Analysis of Virulence Factor Genes

Several of the VFGs detected in this study encode for adhesion, motility, and biofilm formation, allowing bacteria to remain and persist in the wastewater environment and survive WWTP treatment systems, thus managing to escape into the environment via treated wastewater. Mao G et al. evaluated the role of biofilms as a reservoir and vehicle for virulence factors in wastewater, demonstrating that bacteria in this environment increased their biofilm production and that the mobility of virulence factors is crucial for biofilm formation [11].

The present work has some limitations. This study included a reduced number of WWTPs, and the wastewater sampling was only carried out in the influent and effluent. Considering these points, we propose that future work should include a larger number of WWTPs, which would allow us to have geographical representativeness throughout the country, in addition to evaluating the dynamics of bacterial populations, ARGs, plasmids, and VFGs in the different stages of treatment within WWTPs, to evaluate the effectiveness of the elimination in each process.

## 4. Materials and Methods

### 4.1. Study Design and Sample Collection

This descriptive longitudinal study was conducted in Mexico City and Cuernavaca City from August 2021 to September 2022. Samples of raw (100 mL) and treated (200 mL) wastewater were collected in each season of the year; two samples of treated wastewater and two samples of raw wastewater in each included WWTP were collected. Samples were taken at one-month intervals between each sample. The WWTPs studied were the community WWTPs ACA with a 18.95666 N latitude and 99.23411 W longitude in Cuernavaca City and COY with a 19.36392 N latitude and 99.11668 W longitude in Mexico City, the hospital WWTP CAN with a 19.17128 N latitude and 99.06314 W longitude, and a second hospital, NUT, with a single sump with a 19.28814 N latitude and 99.15654 W longitude, from which raw wastewater samples were taken, both located in Mexico City. The samples were transported to the laboratory at 4 °C within less than two hours of collection [78,79].

### 4.2. Wastewater Treatment Plant Characteristics

The design of the three WWTPs (ACA, COY, and CAN) starts with a pretreatment process using a griller that eliminates large materials. Subsequently, the water flow passes through an aeration process mediated by regulating valves in different chambers. The treatment process continues with a sedimentation phase, precipitating the sludge. Tertiary treatment includes granular activated carbon filters, zeolite filters, ultraviolet light (UVL), and calcium hypochlorite tablets in the effluent (only CAN WWTP has tertiary treatment). The NUT hospital has only one sump system (pretreatment).

### 4.3. DNA Extraction

All aliquots collected (100 mL of raw wastewater and 200 mL of treated wastewater) were centrifuged at 5000× *g* for 20 min at 4 °C. The supernatant was decanted and one milliliter of EC lysis solution (1M Tris pH 8.0, EDTA, sodium deoxycholate, N-lauryl sarcosyl, RNAase, lysozyme, and lysostaphin) was added to the pellet and incubated at 37 °C for four hours. Then, the ESP solution (EDTA + N-lauryl sarcosyl + proteinase K) was added and incubated at 50 °C overnight [80]. The samples were purified with the Wizard^®^ Genomic DNA Purification Kit (PROMEGA Corp., Madison, WI, USA) according to the manufacturer’s instructions. DNA was quantified by fluorometry using the Qubit 4 Fluorometer (Thermo Fisher Scientific, Waltham, MA, USA). The DNA samples were stored at −20 °C until sequencing.

### 4.4. Sequencing and Bioinformatics Analysis

The 31 samples were sequenced by Illumina HiSeq (Illumina, Inc., San Diego, CA, USA), with 2 × 150 configuration (21.4 GB). Libraries were performed using the Nextera DNA protocol. The reads are available from NCBI SRA under BioProject **ID1197482**.

Adapters were removed for raw reads and a Q > 20 was considered with Trimmomatic (v0.39) [81]. Quality control statistics were performed with FastQC (v0.12.1) [82]. Metagenome assemblies were performed with IDBA-UD (v1.1) [83,84]. Mapping statistics were performed with Bowtie 2 (v2.5.4) [85]. For functional annotation, the Trinotate (v3.0.1) pipeline was employed [86,87]. Gene abundance was estimated as coverage by mapping reads to contigs using BWA (v0.7.12-r1039) and the coverBed function of bedtools (v2.25.0) [88,89]. Kraken (v2.1.3) was used for taxonomic profiling [90,91]. Alpha and beta diversity analyses were performed with the vegan (v2.4-6) and phyloseq libraries (v1.42.0) [92,93]. The distance matrix for beta diversity was performed with the Bray–Curtis index at the species level. Comparisons between groups were determined with an analysis of similarity (ANOSIM) [93,94]. Abundance plots and histograms were performed with the ggplot2 (v3.5.1) and ggpubr (v0.6.0) library of R [92,95,96].

### 4.5. Analysis of Antimicrobial Resistance Genes and Virulence Genes

The resistome and virulome of the samples were determined using contigs of lengths of >150 bp using ABRicate (v1.0.1) [97] with the Comprehensive Antibiotic Resistance Database (CARD) (v3.2.9) [98] and PlasmidFinder (v2.0) [99]. The identified ARGs were grouped into classes according to the type of antibiotic to which they confer resistance, and quantified by absolute abundance, relative abundance, and normalized abundance expressed as percentages. The virulence factor database (VFDB) and the *E. coli* O-groups and H-types database (EcOH) were used for virulence gene detection [100,101].

## 5. Conclusions

This study reveals the predominant bacterial phyla in wastewater, including clinically relevant bacteria such as *Klebsiella* spp., *E. coli*, *Acinetobacter* spp., and *Pseudomonas* spp., among others, in both community and hospital samples, in function of the treatment and with seasonal variations in abondance. A significant variety of ARGs encoding antibiotics of the last choice were detected in the tested community and hospital wastewater. In addition, this study showed that community wastewater also carries a significant burden of ARGs, suggesting widespread antibiotic use and the spread of AMR beyond hospitals. The results showed that having a tertiary treatment system favors a reduction in ARGs, which could be considered as a proposal aimed at improving WWTP treatment systems in our country. The presence of plasmids carrying ARGs that encode surveillance and reserve antibiotics in wastewater could favor the transfer and spread of resistance between bacteria, which represents an important public health problem. The presence of ARGs, plasmids, and VFGs in treated wastewater may have an ecological impact through their spreading in the environment, through their use in the irrigation of green areas of cities and hospitals and their discharge to municipal sewers. Further analysis of these environments will allow us to generate more scientific evidence to propose public policies for environmental protection and public health. Finally, we consider that the results of this study allowed us to develop the first evidence of the seasonal behavior of bacterial populations, ARGs, plasmids, and VFGs in community and hospital wastewater in our country.

## Data Availability

Data is contained within the article.

## Supplementary Materials

The following supporting information can be downloaded at: https://www.mdpi.com/article/10.3390/ijms26052051/s1.
