# Supplementary material for: Bacterial Communities and Resistance and Virulence Genes in Hospital and Community Wastewater: Metagenomic Analysis
Source: Int J Mol Sci. 2025 Feb 26;26(5):2051. doi: 10.3390/ijms26052051 (PMC11900532; doi:10.3390/ijms26052051)

Supplementary Material

Supplementary Figure S1. Relative abundance of antibiotics resistance genes in community wastewater treatment plants ACA (Pink) and COY (Green)

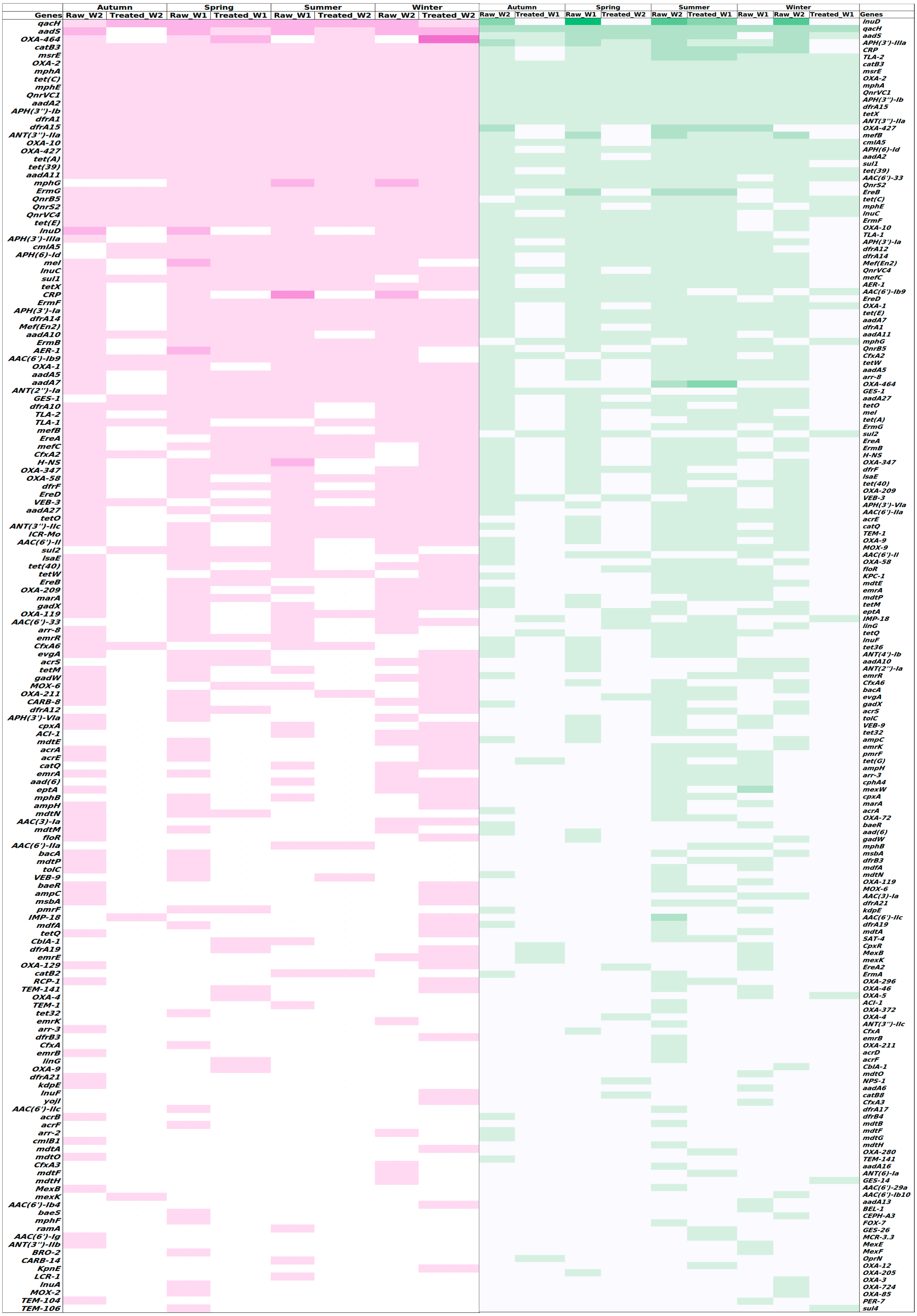

Supplementary Figure S2. Relative abundance of antibiotics resistance genes in hospital wastewater treatment plants CAN (Blue) and NUT (Yellow)

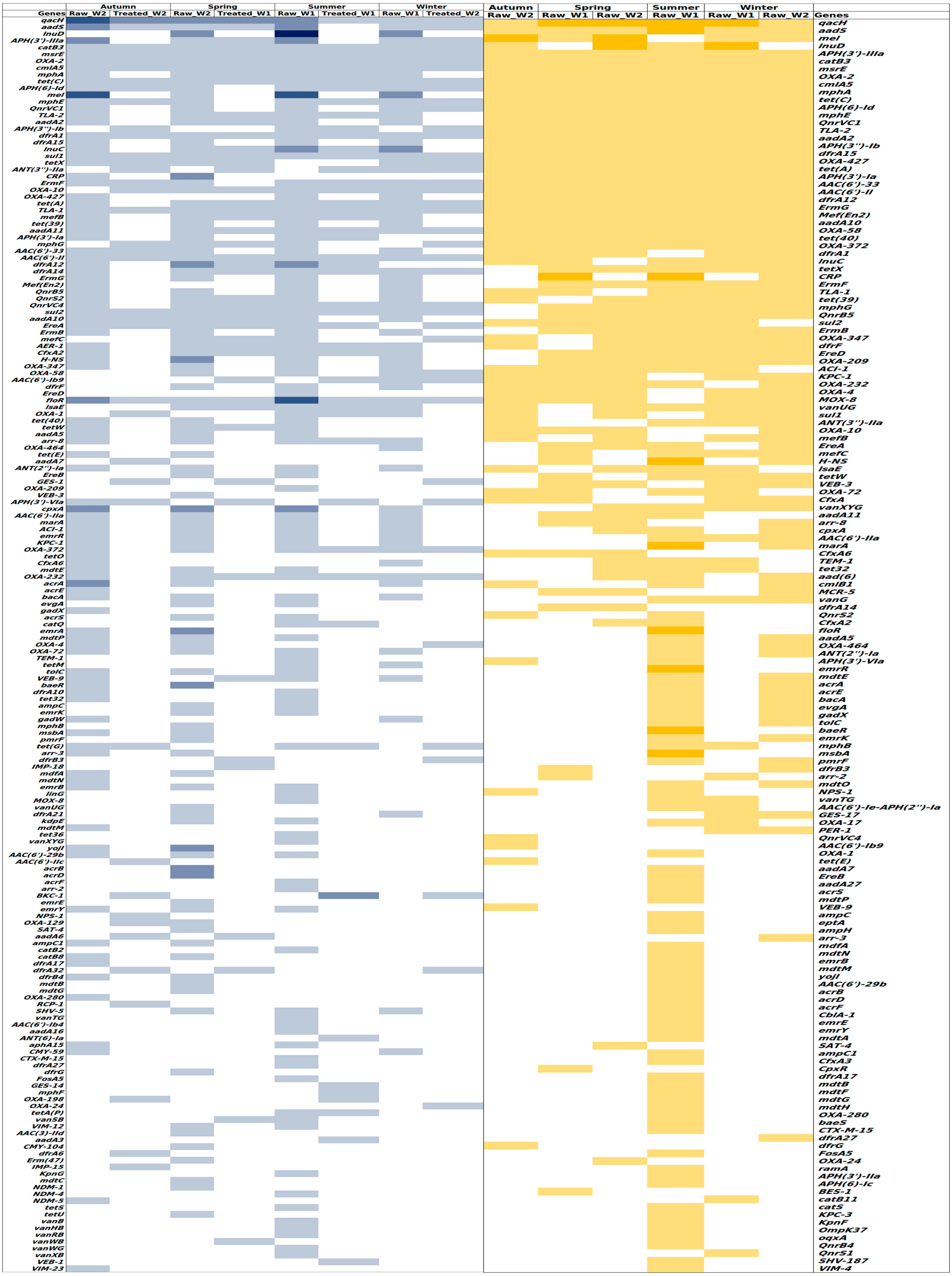

Supplementary Figure S3. Relative abundance of plasmid, in community wastewater treatment plants ACA (Purple) and COY (Green)

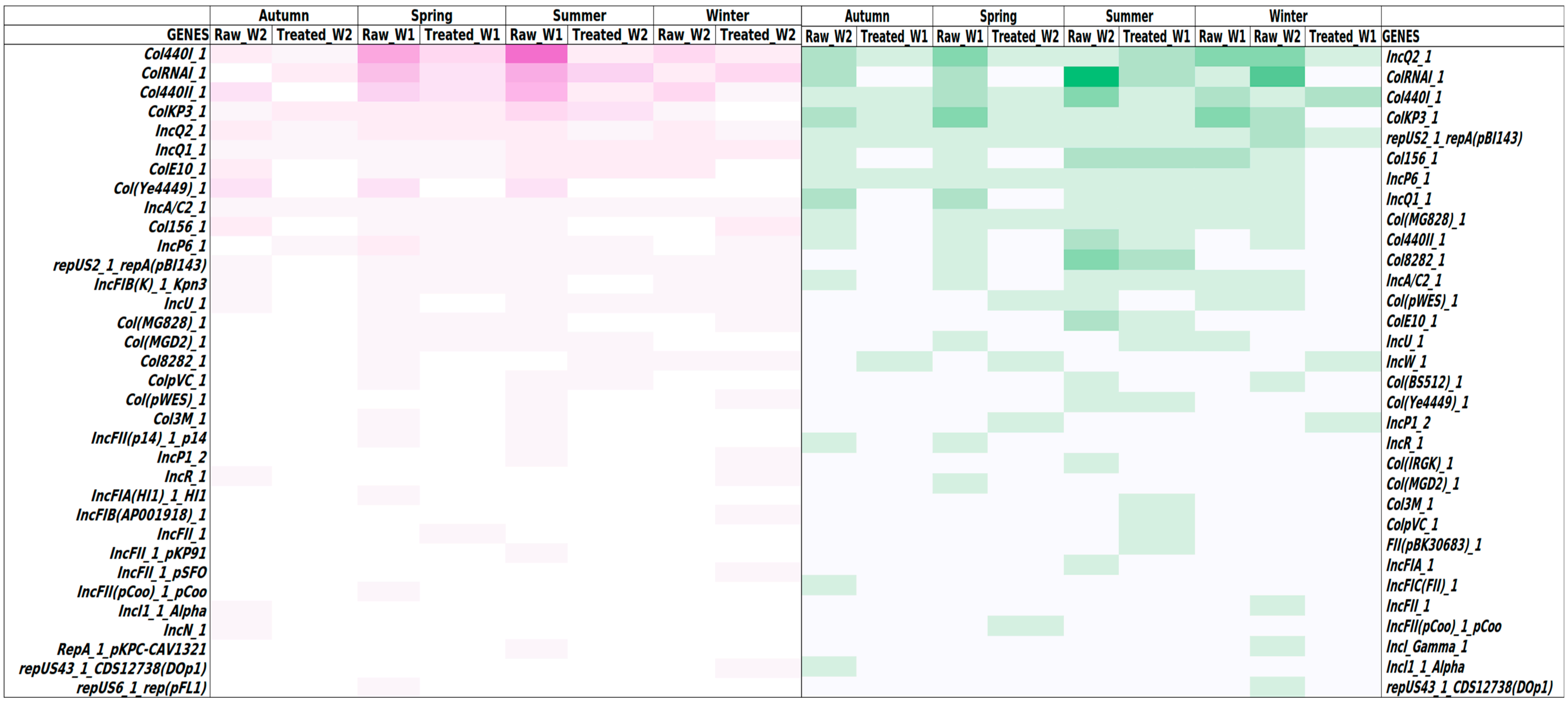

Supplementary Figure S4. Relative abundance of plasmid, in hospital wastewater treatment plants CAN (Blue) and NUT (Pink)

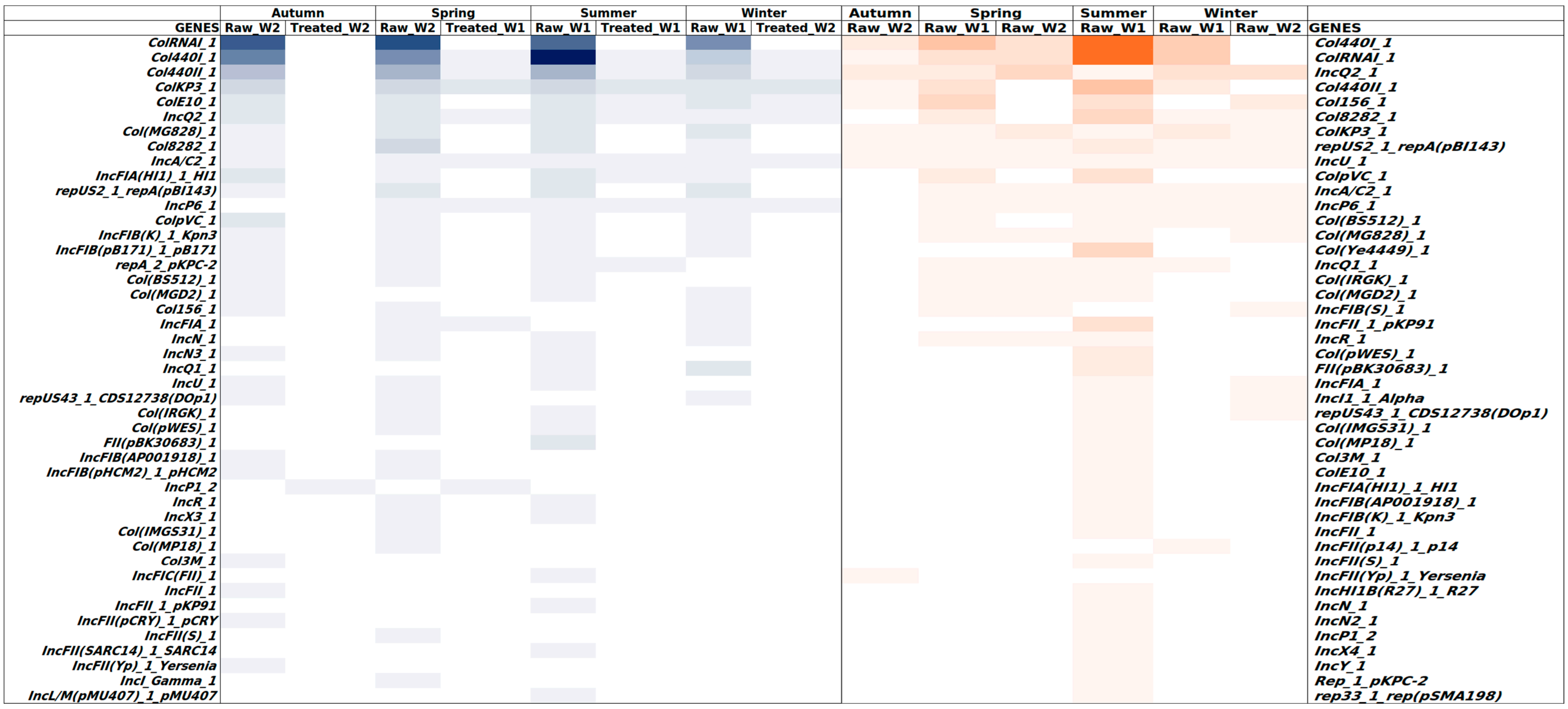

Supplementary Figure S5. Relative abundance of virulence factor genes, in community wastewater treatment plants ACA (Purple) and COY (Green)

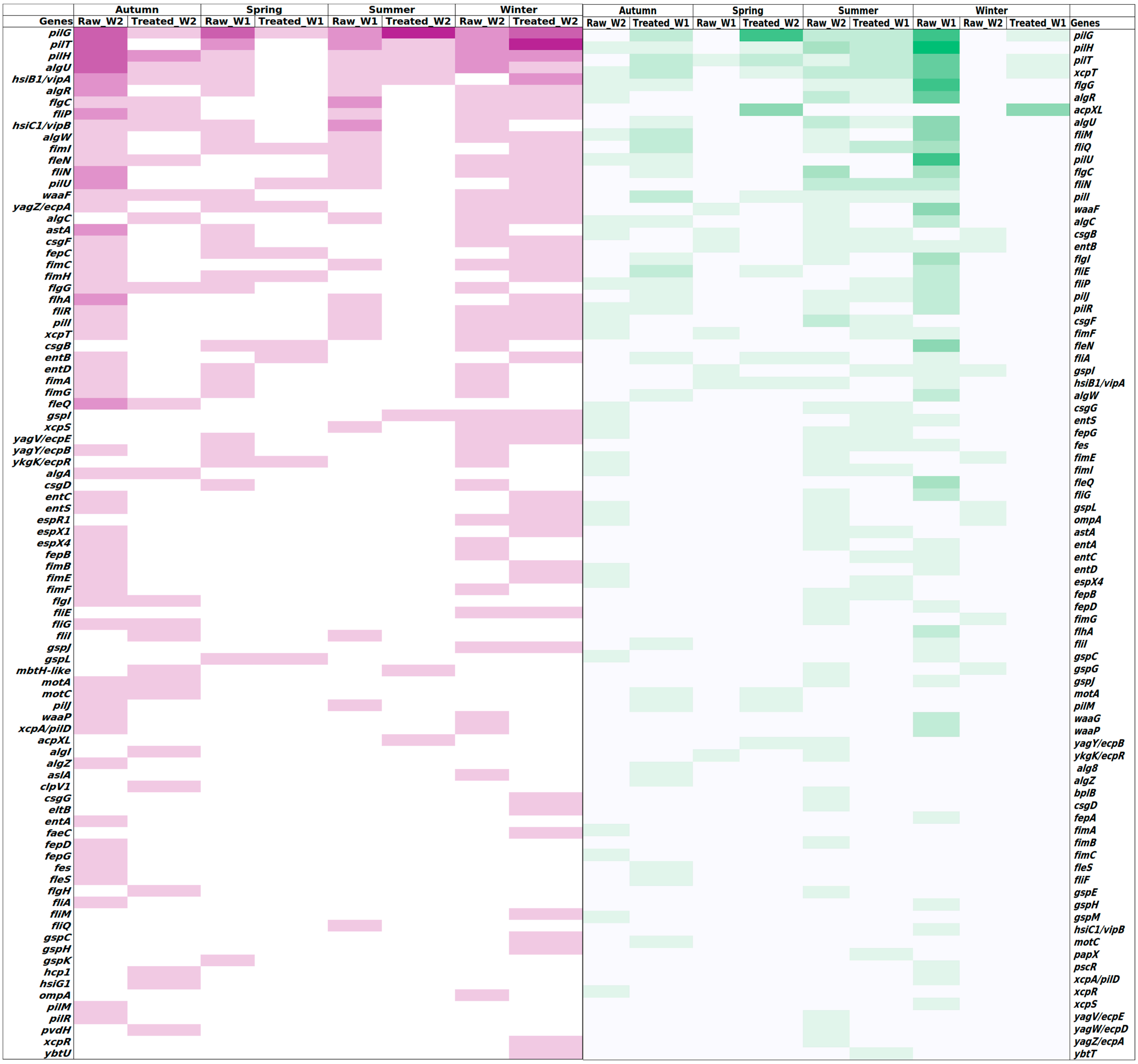

Supplementary Figure S6. Relative abundance of virulence factor genes, in hospital wastewater treatment plants CAN (Blue) and NUT (Pink)

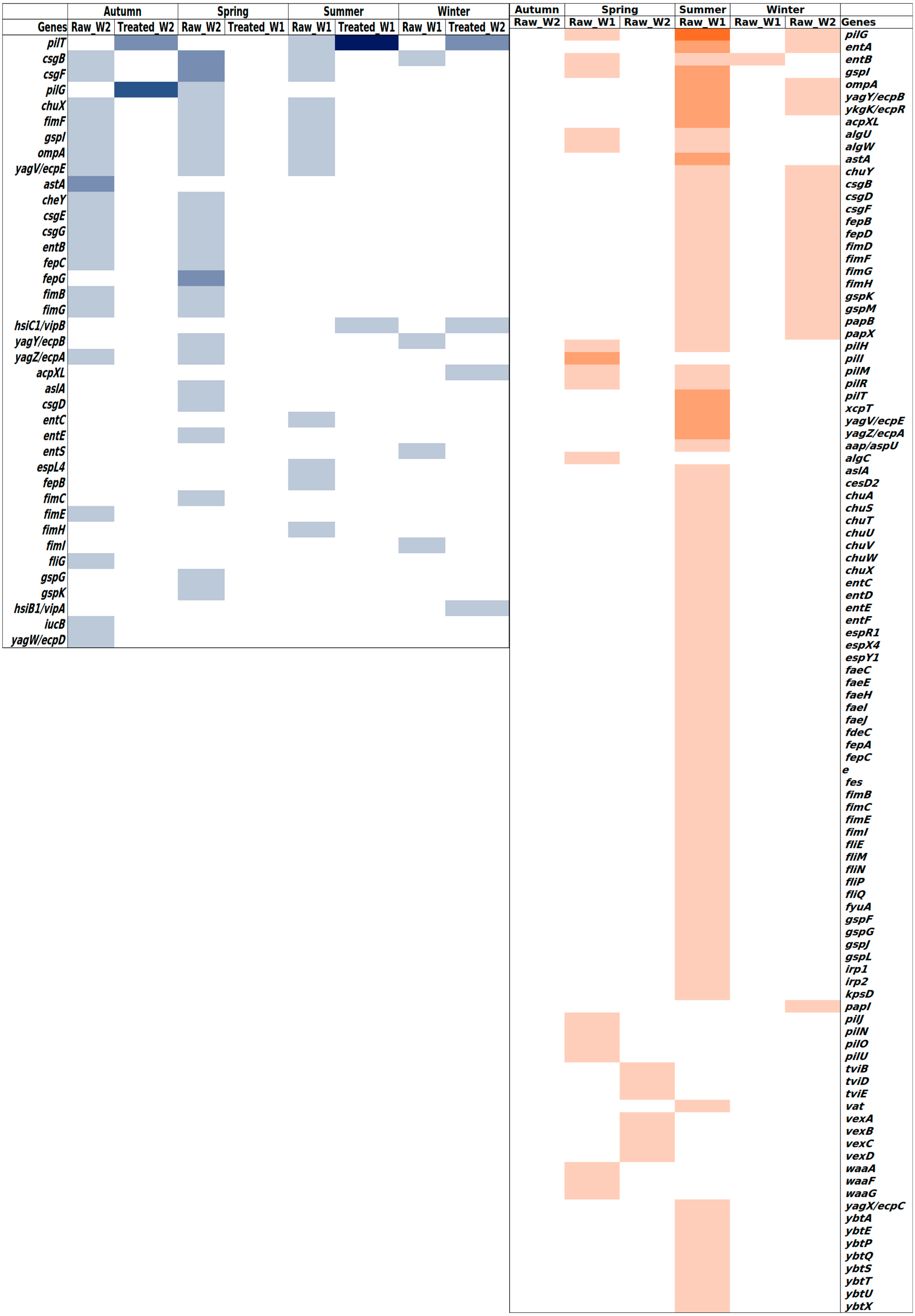

Supplement: Supplementary file 1 [file ijms-26-02051-s001.zip › Supplementary_Material_Fig_S1-S6.pdf]
